# Does pre-angiography Total ST-segment resolution reliably predict spontaneous reperfusion of the infarct-related artery in patients with acute myocardial infarction?

**DOI:** 10.1186/s12872-019-1229-6

**Published:** 2019-11-26

**Authors:** Zongsheng Guo, Xinchun Yang

**Affiliations:** Heart Center, Beijing Chaoyang Hospital, No. 8 workers’ stadium south road, Chaoyang District, Beijing, 100027 China

**Keywords:** ST elevation myocardial infarction, ST resolution, Reperfusion, Percutaneous coronary intervention, Cardiac mortality

## Abstract

**Background:**

ST resolution (STR) after AMI is a non-invasive indicator of IRA reperfusion. We investigated whether pre-angiography STR predicted spontaneous IRA reperfusion in STEMI patients.

**Method:**

Patients with STEMI undergoing primary PCI were recruited. Standard 12-lead ECG tracings were recorded at first medical contact, immediately prior to arterial puncture and 60 min after PCI. STR was classified as total (≥70%; group I), partial (≥30 and < 70%; group II) or none (< 30%; group III). Patients were followed up for 1-year.

**Results:**

The final analysis included 349 patients (*n* = 77, 160 and 112 for groups I, II and III, respectively). Compared with groups I/II, pre-procedural TIMI flow in group III was less frequently grades 2 or 3 (*P* < 0.001). Pre-PCI STR ≥70% was an independent predictor of pre-PCI TIMI-3 flow (OR: 2.8; *P* < 0.001). Pre-PCI STR < 30% was independently associated with pre-PCI TIMI flow 0–2 (OR: 3.1; *P* < 0.001). STR = 35.55% seems to be an optimal cut off for pre-procedural TIMI-3 flow prediction with sensitivity 0.943, specificity 0.456, Youden index 0.399, *P* = 0.027. STR prior to PCI was inversely correlated with 1-year combined CV events rate. STR > 70% may predict a better clinical outcome.

**Conclusions:**

Assessment of STR could potentially be used to stratify risk in patients with STEMI before PCI.

## Background

Reperfusion of the infarct-related artery (IRA) is a critical predictor of prognosis in patients with acute myocardial infarction (AMI) and may be evaluated either angiographically or non-invasively. Thrombolysis in myocardial infarction (TIMI) flow grade estimates epicardial flow by evaluating the flow of contrast material in epicardial coronary arteries during angiography [1]. Post-procedural TIMI flow of the IRA is used for risk stratification of patients with STEMI, [2, 3] but pre-procedural TIMI flow may also be an important predictor of clinical prognosis [4, 5]. In an analysis of randomized trials, pre-procedural TIMI-3 flow was a more powerful prognostic predictor than TIMI-3 flow after angioplasty, [5] underscoring the importance of early flow restoration in patients with STEMI.

The most common non-invasive method for evaluating reperfusion is to analyze the resolution of ischemic ST-segment changes in a series of ECG records. ST-segment changes may be evaluated either as the sum of ST-segment deviations in all leads in a given infarction area or in the single lead with the largest ST deviation [6, 7]. A quick estimation of maximal ST resolution (STR) as a surrogate marker of blood flow provides similar results to analysis of the sum of STR in all leads [8]. It was demonstrated that STR after PCI was a strong and independent predictor of cardiac mortality and recurrence of myocardial infarction (MI) across all spectra of clinical risk, [6, 7, 9] and a lack of STR was even of prognostic value 6 years after the occurrence of AMI [10].

Although pre-procedural TIMI flow is recognized to be a reliable predictor of cardiac mortality in patients with STEMI, the prognostic value of STR preceding primary PCI is still not well established. In this study, we evaluated whether pre-angiography STR reliably predicted spontaneous reperfusion of the IRA in patients with AMI undergoing primary PCI. Furthermore, we investigated whether pre-angiography STR was related to enzymatic infarct size or clinical prognosis.

## Methods

### Study population

From January 2015 to July 2017, we prospectively recruited 366 patients with STEMI using the following inclusion criteria: chest pain lasting > 30 min; ST-segment elevation on the electrocardiogram (ECG) in ≥2 adjacent leads (≥0.2 mV in leads V1–3 and ≥ 0.1 mV in all other leads); the maximal levels of the enzymes, creatine kinase-muscle/brain (CK-MB) and cardiac troponin I (cTnI) were elevated to three times the upper limit of normal [11]; and referral for primary PCI was made within the first 6–12 h after symptom onset [12]. The exclusion criteria were: primary PCI of the left main coronary artery, left bundle branch block, accelerated idioventricular rhythm, ventricular fibrillation or paced rhythm before the procedure (these patients were excluded due to the distinctive ECG pattern observed in such cases); hemodynamic instability before the procedure; previous AMI; previous coronary artery bypass graft (CABG); and refusal to participate in the study. The diagnosis was made and medication, angiography and PCI performed by experienced cardiologists. The study complied with the Declaration of Helsinki. The ethics committee of Beijing Chao-Yang Hospital approved the study protocol, and written informed consent was obtained from each patient.

### Medication and PCI

All patients received an oral loading dose of clopidogrel (600 mg) and aspirin (300 mg) immediately after confirmation of STEMI. A bolus of unfractionated heparin (100 IU/kg) was given intravenously before the procedure. Use of a platelet glycoprotein IIb/IIIa inhibitor before PCI was at the discretion of the interventional cardiologist; if administered, the heparin dose was reduced to 70 IU/kg. PCI was carried out in all patients using a standard method [12]. Other medications used after PCI were given in accordance with current STEMI guidelines [12] and included clopidogrel (75 mg/d) and aspirin (100 mg/d) indefinitely.

The initial laboratory findings, physical examination findings, coronary risk factors, Killip class and left ventricular ejection fraction on echocardiography were acquired by protocol at admission. Total ischemic time (the time from symptom onset to first balloon inflation) was recorded in all patients.

CK-MB and cTnI levels were tested on admission and at 0, 6, 12 and 24 h after PCI. The peak values of CK-MB and cTnI were used as criteria to evaluate enzymatic infarct size [13]. Two-dimensional echocardiography to evaluate left ventricular function was performed on admission and prior to discharge. The 30-day, 6-month and 1-year follow-up was performed by outpatient visit or telephone interview. All-cause death, cardiac death and major cardiovascular (CV) events (including myocardial infarction, revascularization [PCI or CABG], stroke, heart failure and cardiac death) were recorded at each follow-up visit.

### Evaluation of electrocardiographic findings

Standard 12-lead ECG tracings (paper speed, 25 mm/s; amplification, 10 mm/mV) were recorded at three occasions: 1) at first medical contact when STEMI was confirmed and pharmacologic intervention was initiated (on admission or in the ambulance); 2) immediately prior to arterial puncture; and 3) 60 min after PCI. ECG data were evaluated independently by two ECG technicians (each with > 5 years of experience), who were blinded to each other’s analyses and to the clinical characteristics and coronary angiography findings of the patients. ST-segment deviations were calculated for all ECG traces by the two technicians and averaged.

ST-segments were measured at the J point relative to the preceding TP segment (isoelectric line). ST elevation was considered if it was ≥0.25 mV in men aged < 40 years, ≥0.2 mV in men aged > 40 years, or ≥ 0.15 mV in women in leads V2 and V3 and/or ≥ 0.1 mV in other leads, in accordance with current guidelines [12]. The summed ST-segment deviation was calculated by adding the ST-segment elevations in all infarct-related leads and ST-segment depressions in all reciprocal leads [6, 14]. For anterior infarction, the sum of the ST-segment elevations in leads V1 to V6, I and aVL was added to the sum of the ST-segment depressions in leads II, III and aVF. For inferior infarction, the sum of the ST-segment elevations in leads II, III and aVF (and I, aVL, V5 and V6 if present) was added to the sum of the ST-segment depressions in leads V1 to V4. Pre-PCI STR was classified as total (resolution of the initial ST-segment elevation by ≥70%), partial (≥30 and < 70%) or none (< 30%) [8].

### Analysis of angiographic findings

Coronary angiography was performed in all patients. Qualitative and quantitative analyses were made independently by two cardiologists (each with > 8 years of experience), who were blinded to the clinical and ECG characteristics; any inconsistencies were resolved by discussion. The culprit artery, or infarct related artery (IRA) was identified according to the presence of thrombus, total occlusion, or delayed anterograde flow. The flow of the IRA prior to and after PCI was graded as TIMI grade 0 (no perfusion), 1 (penetration without perfusion), 2 (partial perfusion) or 3 (complete perfusion) according to the Thrombolysis in Myocardial Infarction (TIMI) flow classification [15]. Successful PCI was defined as post-procedural TIMI-3 flow in the IRA, with a residual stenosis ≤20%, and absence of clear dissection, thrombus or perforation within the revascularized vessel.

The primary study objective was to evaluate whether pre-angiography STR was predictive of spontaneous reperfusion of the IRA in patients with STEMI undergoing primary PCI. Secondary objectives were to determine whether pre-angiography STR was related to enzymatic infarct size (assessed from the peak cTnI level) or clinical prognosis (assessed from the in-hospital cardiac mortality, 1-year cardiac mortality and the incidence of combined major cardiovascular events including myocardial infarction, revascularization [PCI or CABG], stroke, heart and failure and cardiac death).

### Statistical analysis

All analyses were performed using SPSS 20.0 (IBM Corp., Armonk, NY, USA). Normally distributed, continuous variables are expressed as means ± standard deviations (SDs) and were compared using ANOVA analysis. Categorical variables are expressed as absolute and relative frequencies. and were compared using the chi-squared test. To identify whether pre-procedural STR predicted IRA patency, logistic regression analysis was performed after adjustment for differences in baseline characteristics; odds ratios (ORs) and 95% confidence intervals (95%CIs) were calculated. We evaluated the diagnostic performance of STR at cutoffs of 30 and 70%. An optimal cutoff was expected to have a sensitivity of at least 90%. Correlations between variables were evaluated using Spearman’s correlation coefficients (rS). Survival during follow-up was evaluated by the Kaplan-Meier method and log-rank test. A two-tailed *P*-value < 0.05 was considered statistically significant.

## Results

### Patient characteristics

Between January 2015 and July 2017, 366 of 833 patients with STEMI undergoing primary PCI in our department fulfilled the inclusion criteria and were enrolled in our study. Eight patients underwent primary PCI of the left main coronary artery and were excluded due to the distinctive ECG pattern and prognosis. A further 9 patients failed PCI or underwent CABG and were also excluded due to the distinctive prognosis in such cases. Therefore, a total of 349 patients (257 male and 92 female) were included in the analysis of the pre−/post-procedural ECG, angiography data and follow-up information. Follow-up data at 1 year were obtained from 335 (98%) of the 342 patients who were discharged from hospital alive.

Patients were divided into 3 groups based on pre-angiography STR: group I (STR ≥70%), *n* = 77 (22%); group II (30% ≤ STR<70%), *n* = 160 (46%); and group III (STR < 30%), *n* = 112 (32%). The baseline and procedural characteristics of the study cohort are presented in Table 1. Except for a higher percentage of patients with diabetes mellitus in group III (*P* = 0.03), the patients were comparable between groups with respect to age, gender, risk factors, blood pressure, heart rate, infarct localization, stent implantation, discharge medication and general characteristics of the diseased vessels.

**Table 1:** Clinical characteristics and outcomes stratified by ST resolution (STR)

Variable	STR ≥ 70% *n* = 77 (22%)	30% ≤ STR < 70% *n* = 160 (46%)	STR < 30% *n* = 112 (32%)	*P*
Patient characteristics				
Age (years)	61.2 ± 7.3	62.1 ± 8.4	60.0 ± 7.8	0.12
Male (%)	75.3	74.4	71.4	0.15
Smoking (%)	55.8	52.5	58.0	0.26
Hypercholesterolemia (%)	41.6	37.5	40.2	0.54
Diabetes mellitus (%)	9.1	12.5	21.4	0.03
Renal insufficiency (%)	5.2	5.6	8.0	0.09
Previous infarction (%)	6.5	7.5	8.9	0.14
Heart rate (bpm)	75.6 ± 8.2	72.0 ± 9.3	77.9 ± 7.7	0.87
Killip class > 1 (%)	11.4 ± 3.2	15.8 ± 3.8	19.4 ± 4.6	0.08
Left ventricular ejection fraction (%)	56.6 ± 7.9	52.0 ± 8.2	42.4 ± 7.6	0.02
Home medication (%)				
Aspirin	21.3	24.1	22.6	0.12
Clopidogrel	6.2	5.7	5.2	0.64
Statins	16.4	17.7	15.7	0.55
β blockers	12.4	14.1	13.8	0.76
ACE inhibitors/ATII antagonists	27.3	24.5	25.7	0.14
Ischemia characteristics				
Total ischemic time (min) *	445.6 ± 46.2	481.7 ± 48.4	473.0 ± 41.3	0.07
Anterior MI (%)	45.5	40.0	42.8	0.13
Non-anterior MI (%)	54.5	60.0	57.2	0.45
Maximal cTnI (ng/mL)	0.54 ± 0.08	2.82 ± 0.25	6.03 ± 0.52	< 0.001
Creatine kinase-MB (U/L)	54.0 ± 6.3	168.0 ± 14.4	276.0 ± 26.3	< 0.01
Multi vessel disease (%)	35.0	32.5	33.9	0.41
Initial TIMI flow (%)				
0	14.3	36.3	80.4	< 0.001
1	7.8	9.4	6.3	0.45
2	26.0	26.9	9.8	< 0.001
3	51.9	27.5	3.5	< 0.001
Baseline Rentrop classification (%)				
0–1	62	66	59	0.23
2–3	38	34	41	0.26
Treatment characteristics (%)				
Stent implantation	93.5	95.6	95.5	0.54
Platelet glycoprotein IIb/IIIa inhibitors	42.9	48.8	57.1	0.03
Post-procedural TIMI flow (%)				
0	1.3	1.2	2.7	0.02
1	1.3	4.4	8.0	0.01
2	6.5	9.4	21.4	0.01
3	90.9	85.0	67.9	0.03
Medical treatment at discharge (%)*				
Aspirin	100	100	100	1
Clopidogrel	100	100	100	1
Statins	100	100	100	1
β-blockers	96.1	98.1	94.4	0.42
ACE inhibitor/ATII antagonists	64.9	59.5	65.4	0.56

cTnI: cardiac troponin I; MI: myocardial infarction; STR: ST resolution; TIMI: thrombolysis in myocardial infarction. * interval from symptom onset to balloon dilation. ACE: angiotensin converting enzyme; ATII: angiotensin receptor subtype II; CV: cardiovascular; STR: ST resolution; TIMI: thrombolysis in myocardial infarction

### Myocardial perfusion

**Fig. 1:**
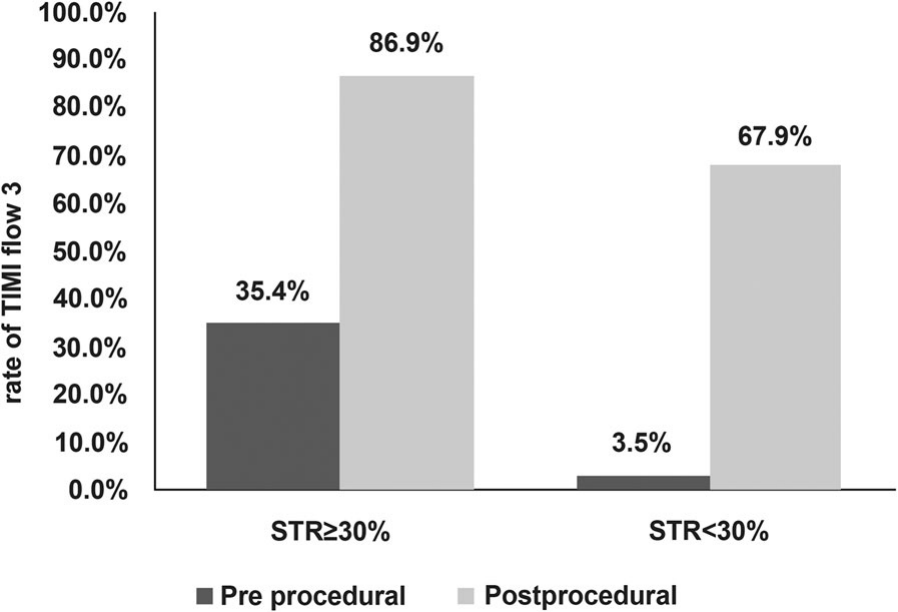
Rates of TIMI-3 flow pre-percutaneous coronary intervention (PCI) and post-PCI in patients with pre-procedural ST resolution ≥30 and < 30%

Peak values of cTnI and CK-MB (used as indicators of enzymatic infarct size) [13] were significantly higher in group III than in the other groups (cTnI, *P* < 0.001; CK-MB, *P* < 0.01; Table 1). Prior to PCI, TIMI-0 flow in the IRA was observed more frequently in group III than in groups I and II (*P* < 0.001), while TIMI-2 and TIMI-3 flow were observed more often in groups I and II than in group III (*P* < 0.001; Table 1, Fig. 1). The interval from symptom onset to balloon dilation did not differ significantly between groups.

**Fig. 2:**
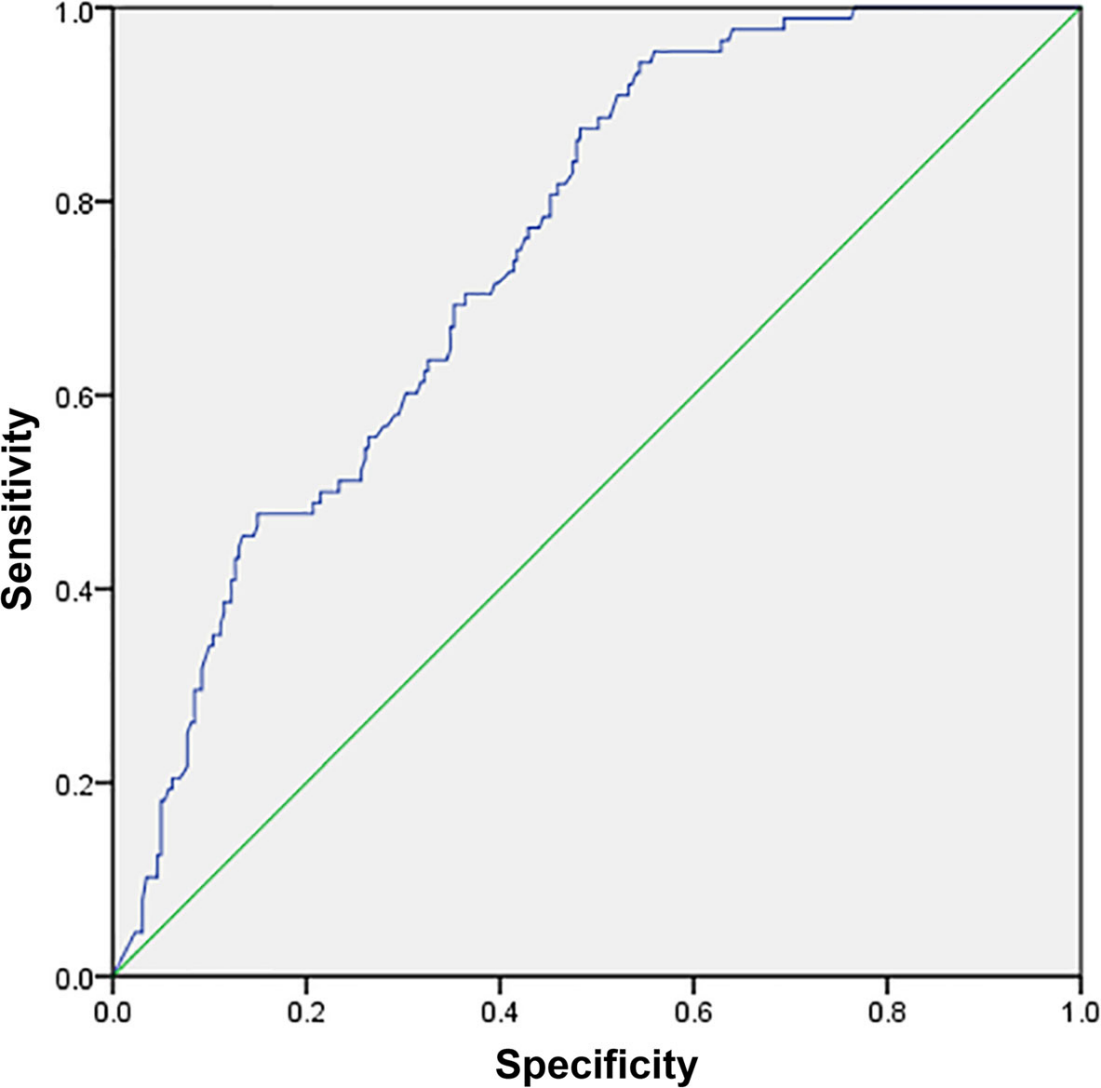
Accuracy of STR to predict pre-procedural TIMI flow 3

Correlation analysis indicated that STR prior to PCI was inversely correlated with impaired TIMI flow (grade 0–2) at initial angiography (rS = − 0.513, *P* < 0.05), peak cTnI value (rS = − 0.484, *P* < 0.01) and peak CK-MB value (rS = − 0.526; *P* < 0.01). Logistic regression identified complete STR (> 70%) prior to emergency angiography as an independent predictor of pre-procedural TIMI-3 flow (OR: 2.8; 95%CI: 1.3–4.8; *P* < 0.001), while non-STR (< 30%) prior to emergency angiography was independently associated with impaired pre-procedural perfusion (TIMI flow 0–2) (OR: 3.1; 95%CI: 1.9–5.0; *P* < 0.001). ROC curve indicated STR = 35.55% an optimal cut off for pre-procedural TIMI-3 flow prediction with sensitivity 0.943, specificity 0.456, Youden index 0.399, *P* = 0.027, It seems more effective than 70% and 30% as a prediction of TIMI-3 flow (Sensitivity 0.995, specificity 0.414, Youden index 0.368 for STR =30%; Sensitivity 0.445, specificity 0.858, Youden index 0.313 for STR =70%;) (Fig. 2, Table 2).

**Table 2:** Accuracy of STR to predict pre-procedural TIMI flow 3

STR	Sensitivity	Specificity	Youden index	*P* value
30%	0.955	0.414	0.368	> 0.05
70%	0.455	0.858	0.313	> 0.05
35.55%	0.943	0.456	0.399	0.027

Stent implantation was comparable among the three groups (Table 1), although the proportion of patients treated with platelet glycoprotein IIb/IIIa inhibitors was higher in group III than in the other groups (*P* = 0.03). Successful recovery of TIMI-3 flow after PCI was less frequent in group III than in groups I and II (*P* = 0.03; Table 1, Fig. 1). Patients in groups I and II had a higher left ventricular ejection fraction before discharge than patients in group III (*P* = 0.02).

### Clinical outcome

**Fig. 3:**
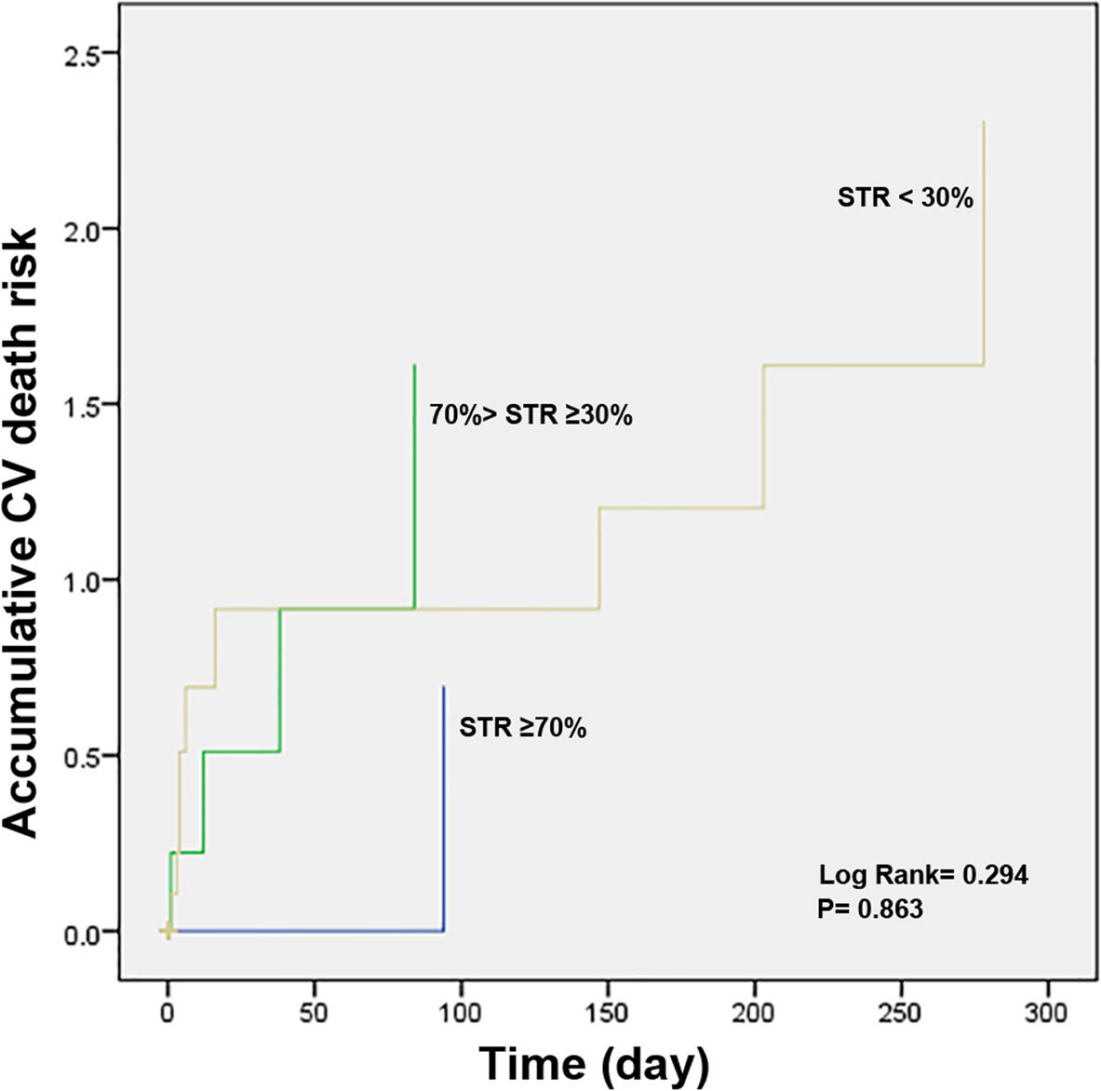
CV death risk of patients with different STR category (Kaplan-Meier curve)

**Fig. 4:**
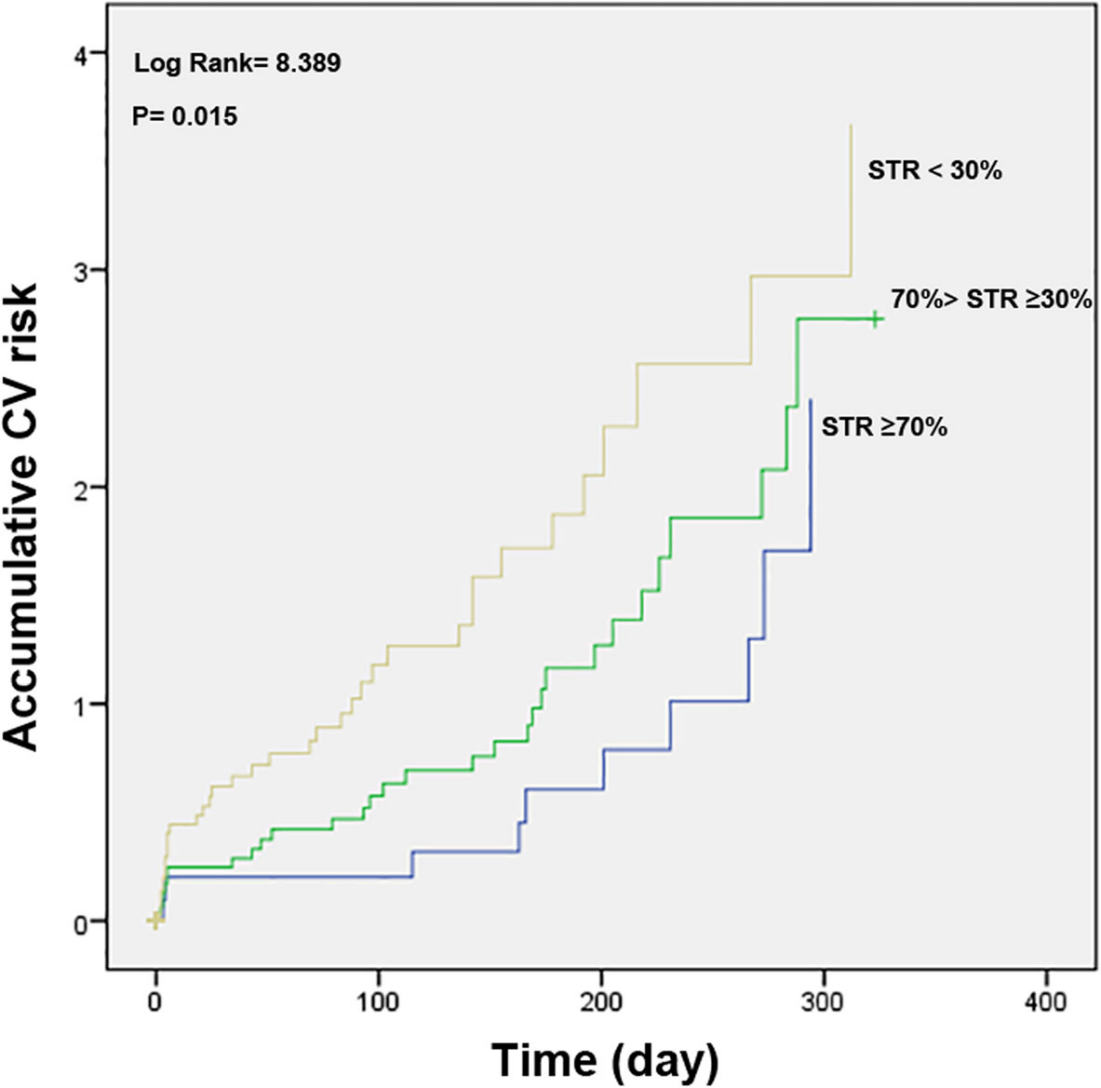
CV risk of patients with different STR category (Kaplan-Meier curve)

Overall in-hospital cardiac mortality was 2.0% (2/160 in group II and 5/112 in group III, no in hospital death in group 1). Medical therapy at discharge was comparable among groups. One-year follow-up data were not available for 7 discharged patients (3 in group III, 3 in group II and 1 in group I). There were additional 10 cardiac deaths (2 in group I, 3 in group II and 5 in group III) in the 1-year follow-up analysis. Cumulative 1-year cardiac mortality rate of all patients was 4.9%, 2.6% in group I, 3.1% in group II, and 8.9% in group III, Log Rank = 8.389. *P* = 0.015 (Fig. 3); 82 out of 349 subjects (23.5%) experienced at least one CV event, 11 in group I (14.3%), 32 in group II (20.0%) and 39 in group III (34.8%), Log Rank = 8.389. P = 0.015 (Fig. 4). Patients with better pre-PCI STR showed improved in-hospital survival, 1-year survival and event-free survival.

## Discussion

Tissue perfusion may be assessed using angiography or electrocardiographic parameters (e.g. STR) [16, 17]. Both angiography and STR can be used to quantify the magnitude of myocardial reperfusion before or after thrombolysis and/or primary PCI. TIMI flow ≥2 prior to thrombolysis or PCI is associated with a smaller enzymatic infarct size and better clinic prognosis independent of the time of reperfusion [4, 18]. Although the relation of STR with enzymatic infarct size [19, 20] and cardiac mortality [8, 21] in patients treated with thrombolytic therapy has been demonstrated by clinical studies, the impact of pre-angiography STR on the prognosis of patients after primary PCI is still being investigated.

Our study investigated the value of pre-procedural ECG for predicting coronary reperfusion and clinical outcome. The average symptom onset-to-balloon time in our patients was 7.8 h. STR prior to PCI was inversely correlated with impaired TIMI flow at initial angiography and with enzymatic infarct size (assessed from peak cTnI and CK-MB values).

Verouden and colleagues concluded that STR is a poor indicator of spontaneous reperfusion [22] and should not be used as a criterion to refrain from immediate coronary angiography in patients with STEMI. We partially agree with this viewpoint. When used as an indicator of spontaneous reperfusion, STR might be influenced by not only reperfusion of the IRA but also the collateral circulation, which could protect the threatened myocardium to some extent. In the absence of collateral flow, the myocardial area at risk (AAR) is the territory distal to the IRA. However, in the presence of collateral flow, the actual infarcted area would be the AAR minus the myocardium salvaged by collateral flow. The actual infarcted area is of great interest in studies evaluating the effectiveness of different reperfusion strategies and is a prognostic factor after STEMI [23, 24]. This concept might partially explain the discrepancy in the predictive accuracy of STR with regard to solo IRA reperfusion. STR reflects cardiac cell physiology and thus is a surrogate marker of blood flow. This might explain why STR probably underestimates the severity of IRA TIMI flow to some extend. In our study a certain cut off STR < 35.55% was an independent predictor of impaired reperfusion (TIMI flow 0–2) with sensitivity 0.943, specificity 0.456, Youden index 0.399, *P* = 0.027. Although the summated ST elevation (sumSTE) at admission appears to be useful in the evaluation of AAR and hence prognosis, [25, 26] we agree with Verouden and colleagues that there is no evidence to support the use of STR as a criterion for not performing immediate coronary angiography in patients with STEMI.

Some investigators have proposed analyzing the residual absolute sumSTE rather than its relative change as a surrogate outcome measure [6, 27]. Some researchers have documented the superiority of residual sumSTE over resSTE in the prediction of cardiac mortality [6, 28]. In our study, STR prior to PCI was inversely correlated with 1-year combined CV events rate. Furthermore, complete STR (> 70%) prior to emergency angiography may predict a better clinical outcome.

## Conclusions

We demonstrated that complete STR prior to emergency angiography was an independent predictor of TIMI flow pre-PCI as well as 1-year cardiac mortality rate and 1-year CV events rate. Clinically, STR may imply reperfusion of the AAR by both the IRA and collateral flow. Our findings support STR as a simple and rapid measure to identify the risk level in patients before PCI. Patients with persistent ST elevation should be given more medical attention due to their high in-hospital and 1-year cardiac mortality rates. Further studies should be carried out in a multi-institution setting with a large sample size to avoid selection bias. In addition, a standardized STR measurement should be developed to increase its reliability and repeatability.

## Data Availability

The datasets used and/or analysed during the current study are available from the corresponding author on reasonable request.

## Abbreviations

AMI: Acute myocardial infarction
CABG: Coronary artery bypass graft
CK-MB: Creatine kinase-muscle/brain
IRA: Infarct-related artery
STR: ST resolution
TIMI: Thrombolysis in myocardial infarction
